# Stereotactic Body Radiotherapy in Contralateral Kidney Oligometastasis From Renal Cell Carcinoma in a Nephrectomized Patient

**DOI:** 10.31486/toj.20.0080

**Published:** 2021

**Authors:** Alessandro Di Marzo, Michelina Casale, Fabio Trippa, Paola Anselmo, Fabio Arcidiacono, Lorena Draghini, Sara Terenzi, Ernesto Maranzano

**Affiliations:** Radiotherapy Oncology Centre, S. Maria Hospital, Terni, Italy

**Keywords:** *Antineoplastic agents*, *carcinoma–renal cell*, *drug therapy*, *neoplasm metastasis*, *radiosurgery*

## Abstract

**Background:** Renal cell carcinoma (RCC) frequently metastasizes to distal organs such as the lungs, abdomen, bones, and brain. Although rare cases of adrenal gland metastasis from RCC have been described, to our knowledge, no cases have reported the use of stereotactic body radiotherapy (SBRT) in contralateral kidney oligometastasis in a nephrectomized patient with RCC.

**Case Report:** We report a rare case of single contralateral renal metastasis from RCC in a 65-year-old female that occurred 1 year after right radical nephrectomy. At diagnosis of relapse, the patient received targeted therapy with sunitinib for 9 consecutive months, resulting in a partial regression of renal metastasis. To preserve the organ and consolidate response, SBRT was administered to the residual mass. Targeted therapy was temporarily discontinued 15 days before and after SBRT. Total SBRT dose was 40 Gy in 5 daily fractions given with volumetric modulated arc and image-guided technique. Three months later, magnetic resonance imaging documented a complete regression of disease, a result that persisted at the last follow-up 19 months after SBRT.

**Conclusion:** The combination of sequential targeted therapy and SBRT provided an excellent outcome in a patient with a solitary kidney who experienced contralateral kidney metastasis from RCC. This treatment approach was well tolerated and controlled the disease.

## INTRODUCTION

Renal cell carcinoma (RCC) is the third most common type of urologic cancer after prostate and bladder cancers, and among patients with initially localized disease who undergo nephrectomy, 30% to 40% develop metastases.^1^ RCC is well known for its unpredictable mode of spread and can metastasize to nearly every organ rapidly or after a delay; it most commonly metastasizes to regional lymph nodes, the lungs, the liver, bones, adrenal glands, the brain, and skin. Metastasis to the contralateral kidney is uncommon, with no reported cases to our knowledge as of August 2020, and the probability of developing an asynchronous contralateral primary cancer is rare. The practical dilemma is differentiating a metastatic deposit from an asynchronous primary tumor in the postnephrectomy residual kidney, with biopsy unable to discriminate between the 2 entities.

Molecular targeted therapy has markedly improved the prognosis of patients with metastatic RCC. The tyrosine kinase inhibitor sunitinib has a reported median progression-free survival of 11.5 months and overall survival of 26 months that can reach 40 months with adequate sequential therapy.^1,2^ Depending on their age and history, patients with metastatic RCC can receive different treatments, including surgery, molecular targeted therapy, and immunotherapy.^3^ While the standard of care for metastatic RCC is systemic therapy, local treatment of metastases remains controversial.

The oligometastatic state is a new disease entity (variously defined with 1 to 5 metastases) that is amenable to curative treatment strategies.^4^ Patients with a controlled primary tumor and oligometastatic disease can be effectively treated with local therapies such as surgery or metastasis-directed radiotherapy.^5^ A surgical series suggested that selected patients with RCC receiving curative-intent metastasectomy survived with a longer disease-free interval compared with patients who had not received metastasectomy.^6^ Another series showed improved survival after resections of multiple limited metastases.^7^ These findings showed that complete metastasectomy in selected patients with oligometastatic RCC can improve survival and quality of life. Stereotactic body radiotherapy (SBRT), a form of locally ablative treatment in patients with a limited number (≤5) of distant metastases, was described in October 2018 at the European Society for Medical Oncology Congress in Munich, Germany, as “a new technology in radiotherapy delivery, allowing for potentially curative treatment in oligometastatic patients.”^8^ The choice between SBRT or surgery should be made on a case-by-case basis and ideally by a multidisciplinary team.

Because RCC is generally considered a radioresistant tumor, conventional fractionated radiotherapy has historically been used on few occasions and only with palliative intent.^9^ However, recent (2019) radiobiologic evidence has shown that tumor radioresistance may be overcome with dose escalation, particularly by increasing the dose per fraction.^10^ SBRT, defined as 1 to 5 treatments of high-dose radiation delivered to tumors, was initially used to manage brain metastases but has also been used for extracranial metastases.^11,12^ SBRT allows administration of external beam accelerated hypofractionated high doses to the metastasis and spares surrounding healthy tissues by a rapid fall of dose outside the target.

We report a rare case of single contralateral renal metastasis from RCC that occurred 1 year after right radical nephrectomy and was treated with targeted therapy and SBRT.

## CASE REPORT

A 65-year-old female developed contralateral kidney metastasis 1 year after left radical nephrectomy for clear cell RCC. The patient was referred to our department in April 2018 and was in overall good health, with a Karnofsky Performance Status score of 100%. Her medical history included diabetes mellitus, arterial hypertension, and hypothyroidism pharmacologically well controlled. Physical signs were as follows: oriented, collaborating, and autonomous walking; no neurologic examination abnormalities; blood pressure 125/70 mmHg and pulse 75/min; no fever; and regular bowel and bladder functions. Routine laboratory tests including complete blood count, renal and liver function tests, and electrolyte count were within normal range. Radical right nephrectomy had been performed in April 2016, and postoperative pathology findings confirmed a localized clear cell RCC grade 2 tumor (pT2N0M0 stage II) according to the American Joint Committee on Cancer 2017 cancer staging.^13^ No recurrence was observed during follow-up until July 2017, when total body computed tomography (CT) revealed a 24-mm enhanced mass in the left kidney in absence of other signs of metastasis in the brain, lungs, liver, abdominal lymph nodes, and bones. The patient refused biopsy, so contralateral kidney metastasis rather than asynchronous primary tumor was diagnosed based on the short time from nephrectomy—14 months—and the peripheral location of the lesion.

**Figure 1.**
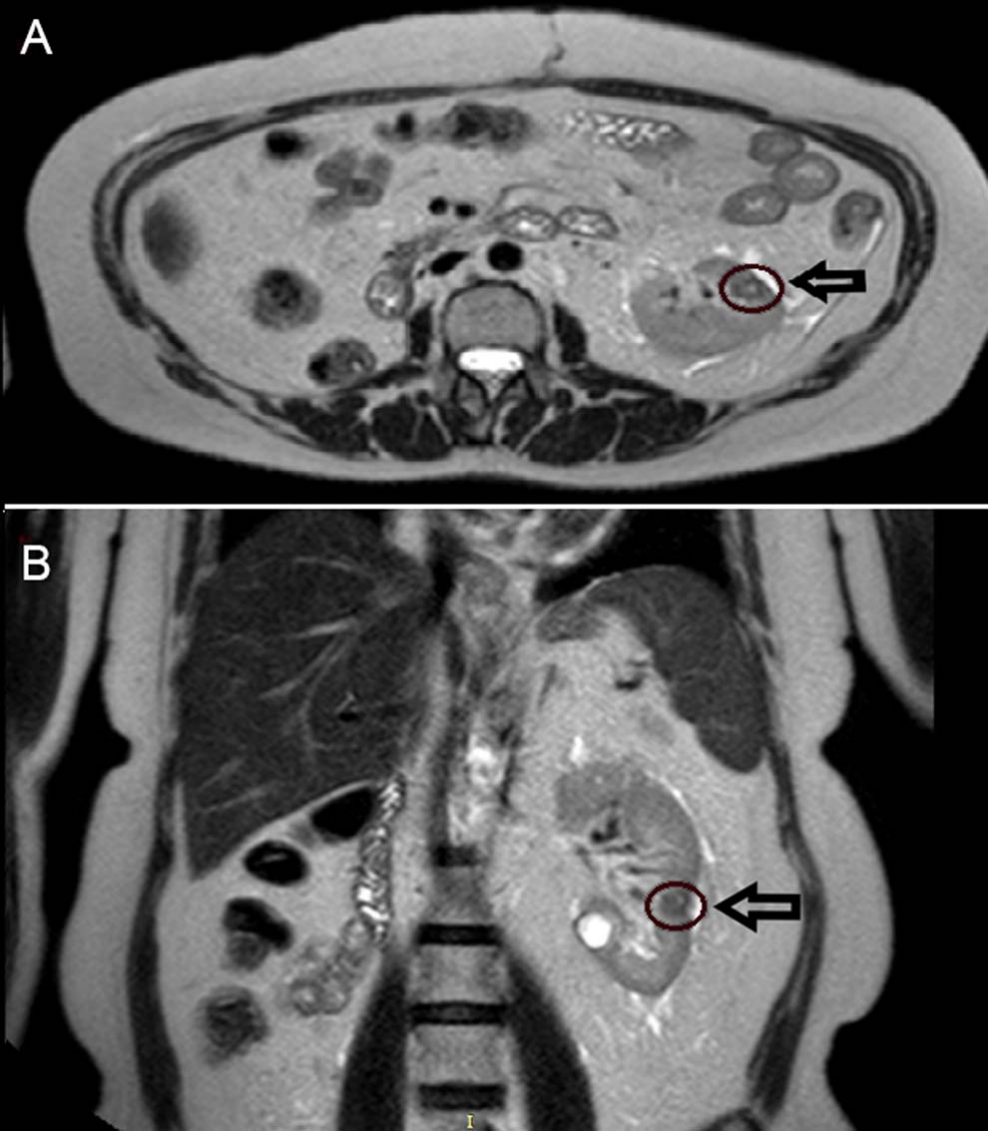
T2-weighted (A) axial and (B) coronal magnetic resonance images show the left kidney metastasis (arrows/circles).

The patient started targeted therapy with sunitinib, administered at a dose of 50 mg orally once daily for 4 weeks of each 6-week cycle. Nine months later, the kidney metastasis had decreased from 24 mm to 12 mm. The patient's case was discussed by the urologic multidisciplinary group of our hospital, and SBRT indication for the kidney residual lesion (Figure 1) was shared among all specialists with the intent to consolidate the response obtained with targeted therapy. Targeted therapy was temporarily discontinued 15 days before and after SBRT. To better define the target, the patient was studied with magnetic resonance imaging (MRI).

**Figure 2.**
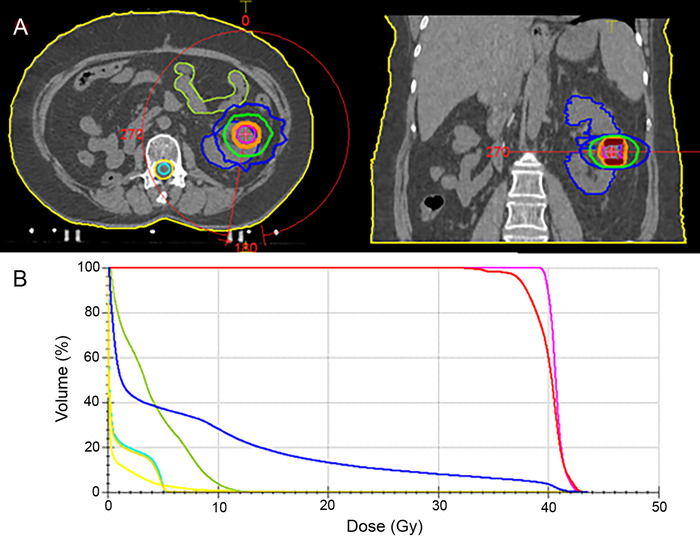
**Treatment planning in (A) axial and coronal views shows metastasis-directed stereotactic body radiotherapy volumes: orange line corresponds to isodose of 95%, green line to isodose of 50%, and blue line to isodose of 30%. In the dose-volume histogram (B), the pink line corresponds to the planning target volume, the red line to the clinical target volume, the blue line to the left kidney, the light green line to the small bowel, the green line to the spinal cord, and the yellow line to cord planning rescue volume.** (A color version of this graphic is available online at https://doi.org/10.31486/toj.20.0080.)

A planning CT scan was acquired with the patient in supine position within an immobilization device, and a specially designed coordinate-system (body frame) was used for the exact localization of the tumor. An abdominopelvic thermoplastic mask was used to minimize setup motion. MIMvista, version 6.6 (MIM Software Inc) was used for volume contouring and image fusion of MRIs with planning CT. Gross tumor volume (GTV) was defined as the radiologically visible lesion volume in contrast-enhancing T2-weighted MRI sequences. Clinical target volume (CTV) was coincident with GTV, and planning target volume (PTV) was GTV/CTV plus an additional 4 mm in all directions. The spinal cord, a safety margin from the cord (cord planning rescue volume), left kidney, and small bowel were delineated as organs at risk (Figure 2).

SBRT was administered using the volumetric modulated arc therapy technique in absence of free-breathing or breath-hold techniques, designed with the Monaco Treatment Planning system (Elekta AB) via the Monte Carlo algorithm. Treatment was delivered using the Elekta Synergy linear accelerator with 6-MV x-ray beam energy without a flattening filter free mode and a single arc of 340° rotating clockwise from 190° to 170°. The statistical uncertainty of the Monte Carlo algorithm was 1%, and the final dose was calculated with a grid resolution of 2 mm. The prescription dose was a total of 40 Gy in 5 fractions every consecutive day, with 95% of the PTV receiving at least 95% of the prescription dose. Dose constraints adopted were those suggested by Hanna et al (ie, Dmean was 40 Gy; V10Gy for the solitary kidney was 28%).^14^ The PTV and organs at risk doses are shown in the Table.

**Table.** Stereotactic Body Radiotherapy Doses

Area	Parameter	Administered Dose, Gy
Planning target volume	Prescription dose	40
Planning target volume	V95%	38
Organs at risk		
Spinal cord	Dmax	5.3
Left kidney	Mean dose	7.7
Small bowel	Dmax	12.2

Note: The planning target volume is the gross tumor volume/clinical target volume plus an additional 4 mm in all directions.

Dmax, maximum dose; V95%, volume of planning target volume receiving 95% of the prescription dose.

**Figure 3.**
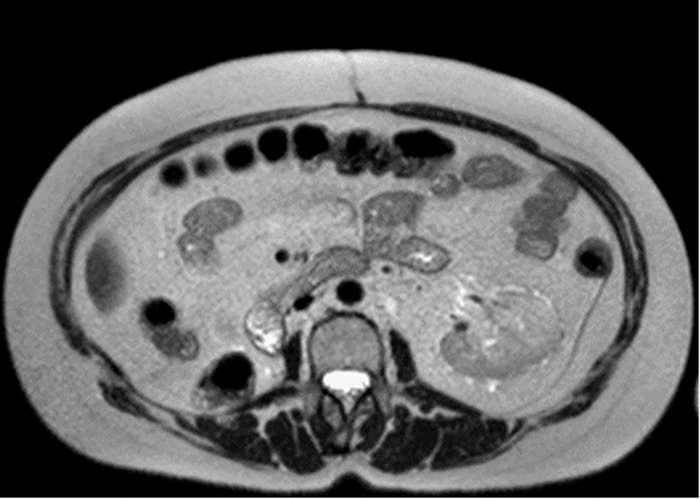
T2-weighted axial magnetic resonance image shows complete response to stereotactic body radiotherapy.

The patient did not receive premedications and had no acute side effects during treatment. No renal function test modification was observed 1 week after SBRT. Creatinine and creatinine clearance values pre-SBRT and post-SBRT remained stable (1.4 mg/dL and 38 mL/min, respectively). To prevent possible CT iodine contrast nephrotoxicity, radiologic follow-up with MRI was done 3 months after SBRT and every 4 months subsequently. At last follow-up 19 months after SBRT, MRI showed a complete response; the treated lesion was no longer detectable (Figure 3).

## DISCUSSION

In metastatic RCC, systemic therapy is the cornerstone of treatment. Targeted therapy with tyrosine kinase inhibitors and checkpoint inhibitors has been established as effective treatment for metastatic RCC, but only a minority of patients achieve complete response after targeted therapy.^15^ Additional approaches are necessary to improve these agents’ efficacy. One strategy relies on the combination of targeted therapy with radiotherapy, thus leading to increased sensitivity of RCC to the effect of ionizing radiation because of the synergy between these modalities.^16^ In several clinical studies, SBRT was shown to be highly effective in controlling bone and lung metastases from RCC.^17^ However, limited data are available on SBRT effectiveness in RCC affecting other organs. In a prospective phase 2 trial, Svedman et al established that SBRT for patients with primary and metastatic RCC provides a high rate of local control (98%).^18^ Wersäll and colleagues showed a local control rate of 90% after SBRT of metastatic RCC to the lung, renal bed, lymph nodes, and adrenal glands.^19^ Altoos et al, analyzing the response rate of SBRT compared to conventional fractionated radiotherapy for thoracic, abdominal, skin, and soft tissue RCC lesions, reported control rates at 12 and 24 months, respectively, of 100% and 93.41% for SBRT vs 62.02% and 35.27% for conventional fractionated radiotherapy.^20^ In a retrospective multicenter Italian study investigating the outcome of treatment with SBRT in 57 nonbrain secondary lesions diagnosed in 48 patients with metastatic RCC, Buti et al reported a 1-year local control rate of 87.7% with SBRT, and 18 patients (37.5%) permanently interrupted systemic therapy after SBRT.^21^ These studies suggest that SBRT may be more effective in controlling RCC lesions than lower dose conventional radiotherapy.

The radioresistance of RCC can be overcome by radiobiologic benefits deriving from hypofractionation and the single higher doses administered with SBRT. In particular, SBRT in a hypervascular tumor such as RCC leads to endothelial injury and antiangiogenic effects.^16^ Thus, in the metastatic setting, and more specifically in the oligometastatic phase with low tumor burden, SBRT could represent a promising local therapeutic option in combination with new drugs. In our rare case of RCC in an already nephrectomized patient with contralateral kidney metastasis partially responsive to 1 year of targeted therapy, we decided to pursue a radical intent by administering SBRT on the residual kidney lesion. In a postsurgical solitary kidney scenario, the critical issue was to deliver an ablative dose to the tumor while sparing functional residual nephrons from radiation injury. The SBRT technique, with hypofractionated radiotherapy, high-precision irradiation, and daily target verification by cone beam CT, allowed us to reach the goal without impairment of patient kidney function.

## CONCLUSION

Although we are conscious that “a swallow does not make spring,” the rarity of the event described in our case report shows the feasibility and effectiveness of SBRT combined with targeted therapy for kidney oligometastasis from RCC in a patient with a solitary kidney.

## Notes

*This article meets the Accreditation Council for Graduate Medical Education and the American Board of Medical Specialties Maintenance of Certification competencies for Patient Care and Medical Knowledge.*
